# Supplementary material for: Monolayer platform to generate and purify primordial germ-like cells in vitro provides insights into human germline specification
Source: Nat Commun. 2023 Sep 14;14:5690. doi: 10.1038/s41467-023-41302-w (PMC10502105; doi:10.1038/s41467-023-41302-w)
Supplement: Supplementary file 1 — Supplementary Information [file 41467_2023_41302_MOESM1_ESM.pdf]

Fig. S1

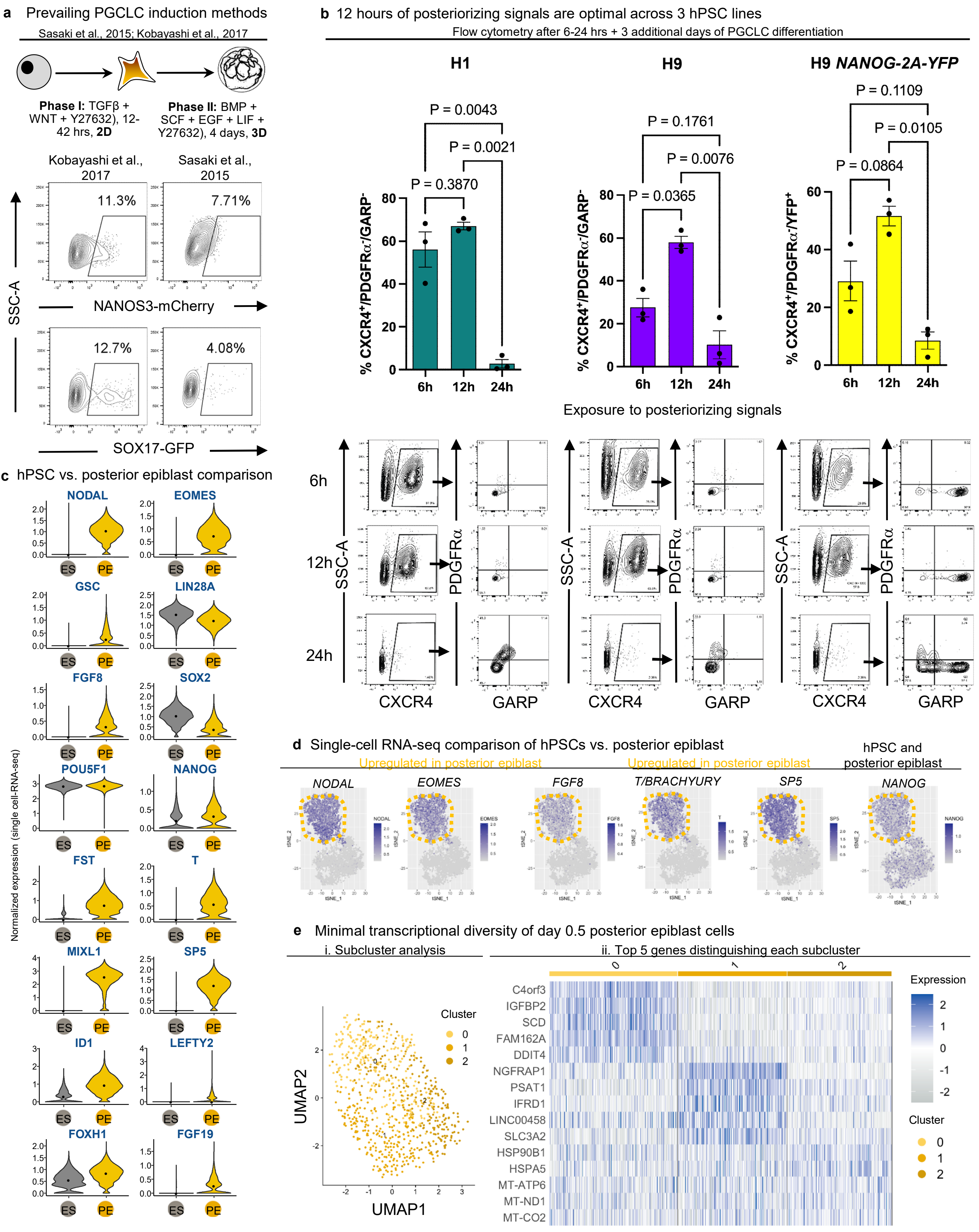
a Prevailing PGCLC induction methods
Sasaki et al., 2015; Kobayashi et al., 2017
Phase I: TGFβ + WNT + Y27632), 12-42 hrs, 2D
Phase II: BMP + SCF + EGF + LIF + Y27632), 4 days, 3D
Kobayashi et al., 2017
Sasaki et al., 2015
11.3%
7.71%
NANOS3-mCherry
12.7%
4.08%
SOX17-GFP
SSC-A
b 12 hours of posteriorizing signals are optimal across 3 hPSC lines
Flow cytometry after 6-24 hrs + 3 additional days of PGCLC differentiation
H1
H9
H9 NANOG-2A-YFP
P = 0.0043
P = 0.0021
P = 0.3870
P = 0.1761
P = 0.0076
P = 0.0365
P = 0.1109
P = 0.0105
P = 0.0864
% CXCR4+/PDGFRα-/GARP-
% CXCR4+/PDGFRα-/YFP+
6h
12h
24h
Exposure to posteriorizing signals
SSC-A
PDGFRα
CXCR4
GARP
c hPSC vs. posterior epiblast comparison
NODAL
EOMES
GSC
LIN28A
FGF8
SOX2
POU5F1
NANOG
FST
T
MIXL1
SP5
ID1
LEFTY2
FOXH1
FGF19
ES
PE
Normalized expression (single cell-RNA-seq)
d Single-cell RNA-seq comparison of hPSCs vs. posterior epiblast
Upregulated in posterior epiblast
Upregulated in posterior epiblast
hPSC and posterior epiblast
NODAL
EOMES
FGF8
T/BRACHYURY
SP5
NANOG
tSNE_1
tSNE_2
e Minimal transcriptional diversity of day 0.5 posterior epiblast cells
i. Subcluster analysis
ii. Top 5 genes distinguishing each subcluster
Cluster
0
1
2
UMAP1
UMAP2
C4orf3
IGFBP2
SCD
FAM162A
DDIT4
NGFRAP1
PSAT1
IFRD1
LINC00458
SLC3A2
HSP90B1
HSPA5
MT-ATP6
MT-ND1
MT-CO2
Expression
2
1
0
-1
-2
Cluster
0
1
2

Figure S1. Further characterization of WNT signaling and candidate posterior epiblast cells, related to Fig. 1

a. Flow cytometry analysis of fluorescent reporter expression during differentiation of WIS1 *NANOS3-mCherry* hESCs and H9 *SOX17-GFP* hESCs differentiated using previously-published protocols (Kobayashi et al., 2017; Sasaki et al., 2015).
b. 6h, 12h, 24h WNT activation in H1, H9, and H9 NANOG YFP. Graphs represent mean with SEM calculated over 3 independent experiments. Below are the FACS plots for each cell line analyzed by CXCR/PDGFRa/GARP staining. N=3, Two-way ANOVA, Tukey's multiple testing correction. Source data are provided as a Source Data file.
c. hPSCs (ES) vs posterior epiblast (PE) marker analysis from single-cell RNA-seq.
d. Single-cell RNA-seq comparison between hPSCs and posterior epiblast.
e. UMAP clustering of posterior epiblast (Day 0.5) cells.

Fig. S2

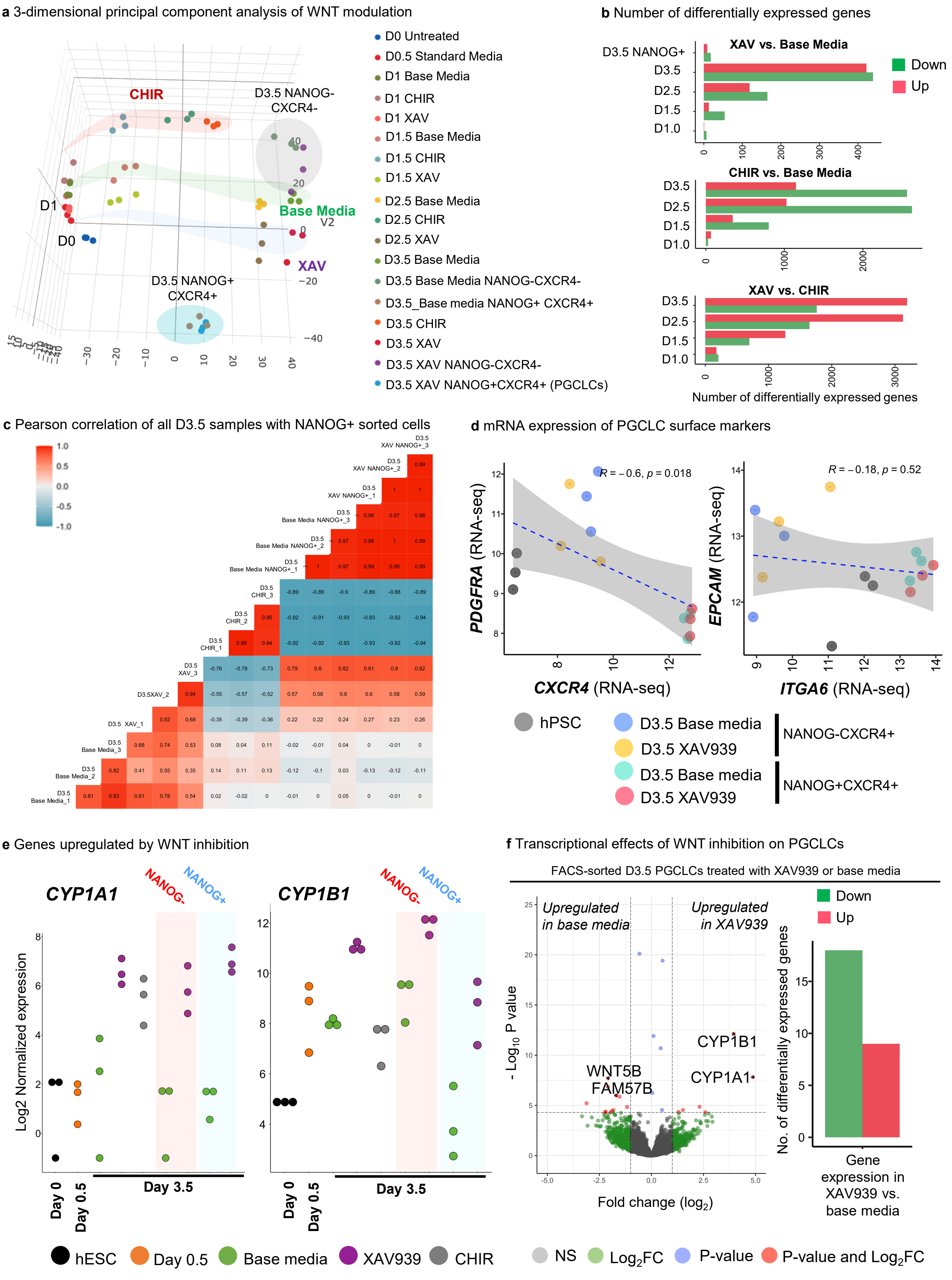
a 3-dimensional principal component analysis of WNT modulation
CHIR
D3.5 NANOG-
CXCR4-
D1
Base Media
V2
D0
XAV
D3.5 NANOG+
CXCR4+
D0 Untreated
D0.5 Standard Media
D1 Base Media
D1 CHIR
D1 XAV
D1.5 Base Media
D1.5 CHIR
D1.5 XAV
D2.5 Base Media
D2.5 CHIR
D2.5 XAV
D3.5 Base Media
D3.5 Base Media NANOG-CXCR4-
D3.5_Base media NANOG+ CXCR4+
D3.5 CHIR
D3.5 XAV
D3.5 XAV NANOG-CXCR4-
D3.5 XAV NANOG+CXCR4+ (PGCLCs)
b Number of differentially expressed genes
XAV vs. Base Media
D3.5 NANOG+
D3.5
D2.5
D1.5
D1.0
Down
Up
CHIR vs. Base Media
XAV vs. CHIR
Number of differentially expressed genes
c Pearson correlation of all D3.5 samples with NANOG+ sorted cells
d mRNA expression of PGCLC surface markers
R = −0.6, p = 0.018
R = −0.18, p = 0.52
PDGFRA (RNA-seq)
CXCR4 (RNA-seq)
EPCAM (RNA-seq)
ITGA6 (RNA-seq)
hPSC
D3.5 Base media
D3.5 XAV939
NANOG-CXCR4+
D3.5 Base media
D3.5 XAV939
NANOG+CXCR4+
e Genes upregulated by WNT inhibition
CYP1A1
CYP1B1
NANOG-
NANOG+
Log2 Normalized expression
Day 0
Day 0.5
Day 3.5
hESC
Day 0.5
Base media
XAV939
CHIR
f Transcriptional effects of WNT inhibition on PGCLCs
FACS-sorted D3.5 PGCLCs treated with XAV939 or base media
Upregulated in base media
Upregulated in XAV939
WNT5B
FAM57B
CYP1B1
CYP1A1
- Log10 P value
Fold change (log2)
NS
Log2FC
P-value
P-value and Log2FC
Down
Up
No. of differentially expressed genes
Gene expression in XAV939 vs. base media

Figure S2 (related to Fig.2): Bulk RNA-seq analysis of samples with and without WNT pathway manipulation.

a. 3D PCA of first three PC components of all bulk RNA-seq samples, with WNT inhibition (XAV939), WNT activation (CHIR) or no manipulation (Base media), across different time points in PGCLC monolayer differentiation.
b. Number of differentially expressed genes comparing different conditions, cut-off at Fold-change of at least 2. Statistical test - Wald (two-tailed test) with Benjamini-Hochberg multiple testing correction, n=3 biological replicates/group. Related to Fig.2.
c. Pearson correlation of NANOG+ PGCLCs with Base media vs. XAV939 vs. CHIR treated Day 3.5 samples. Related to Fig. 2d.
d. Surface marker expression combinations and corresponding Pearson correlation for *PDGFRA* vs. *CXCR4*, or *EPCAM* vs. *ITGA6* in hESC vs. Day 3.5 differentiated NANOG+ PGCLCs vs. NANOG- contaminating cells. Statistical test - Pearson correlation with (2-tailed) 95% confidence interval (error band).
e. Expression of selected genes enriched in NANOG+ cells from XAV treatment (*CYB1A1* and *CYP1B1).* Related to Fig. 8B.
f. Differential gene expression comparison between sorted NANOG+ PGCLCs from Base media vs. XAV939 treated cells. cut-off at Fold-change of at least 2. Statistical test - Wald (two-tailed test) with Benjamini-Hochberg multiple testing correction, n=3 biological replicates/group. Abbreviations: FC- fold change; padj. - adjusted pvalue

Fig. S3

a Prolonged WNT activation leads to sustained primitive streak marker expression

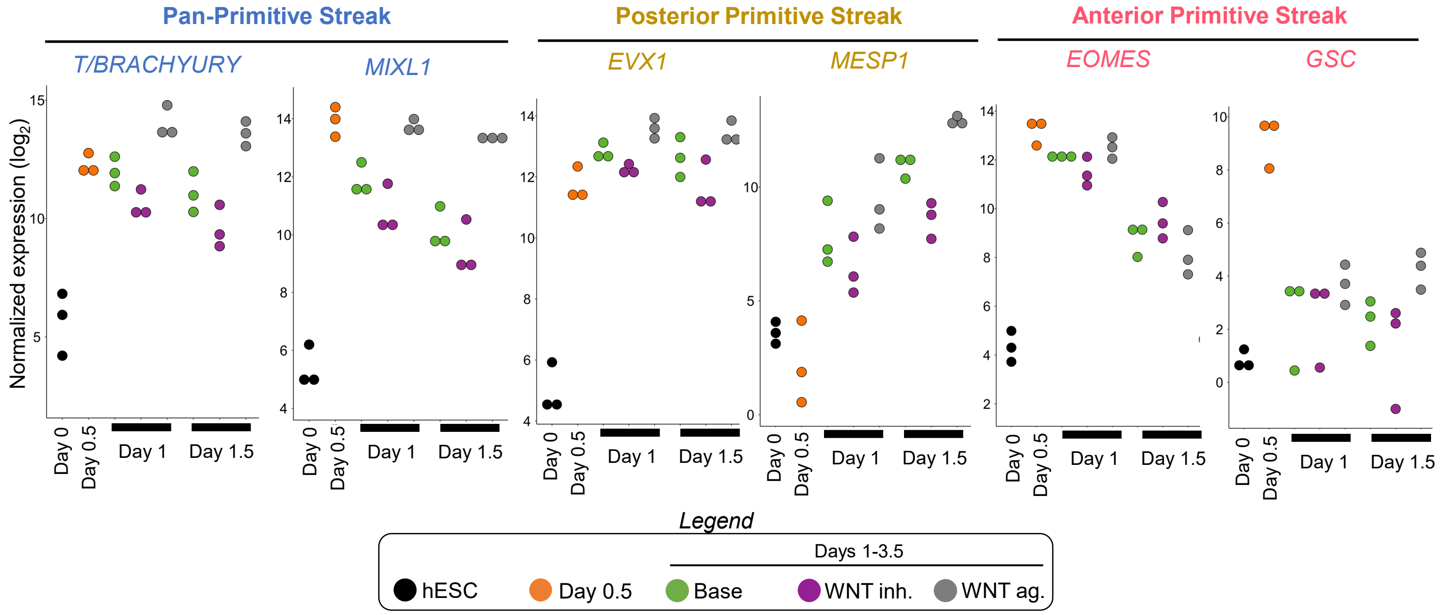

b Prolonged WNT activation strongly blocks PGCLC specification

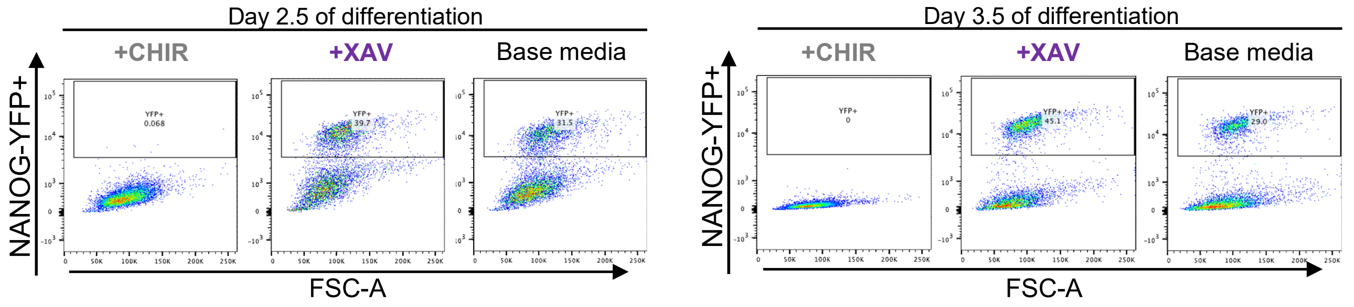

Figure S3 (related to Fig. 2b): WNT activation promotes Primitive streak marker expression.

a. Expression levels of Pan-primitive streak, posterior primitive streak and anterior primitive streak markers under Base media or XAV939 or CHIR99021 treatment.
b. Representative FACS plots of PGCLC sorted cells (NANOG-YFP+) from different treatment conditions at Day 2.5 and Day 3.5. Source data are provided as a Source Data file.

Fig. S4

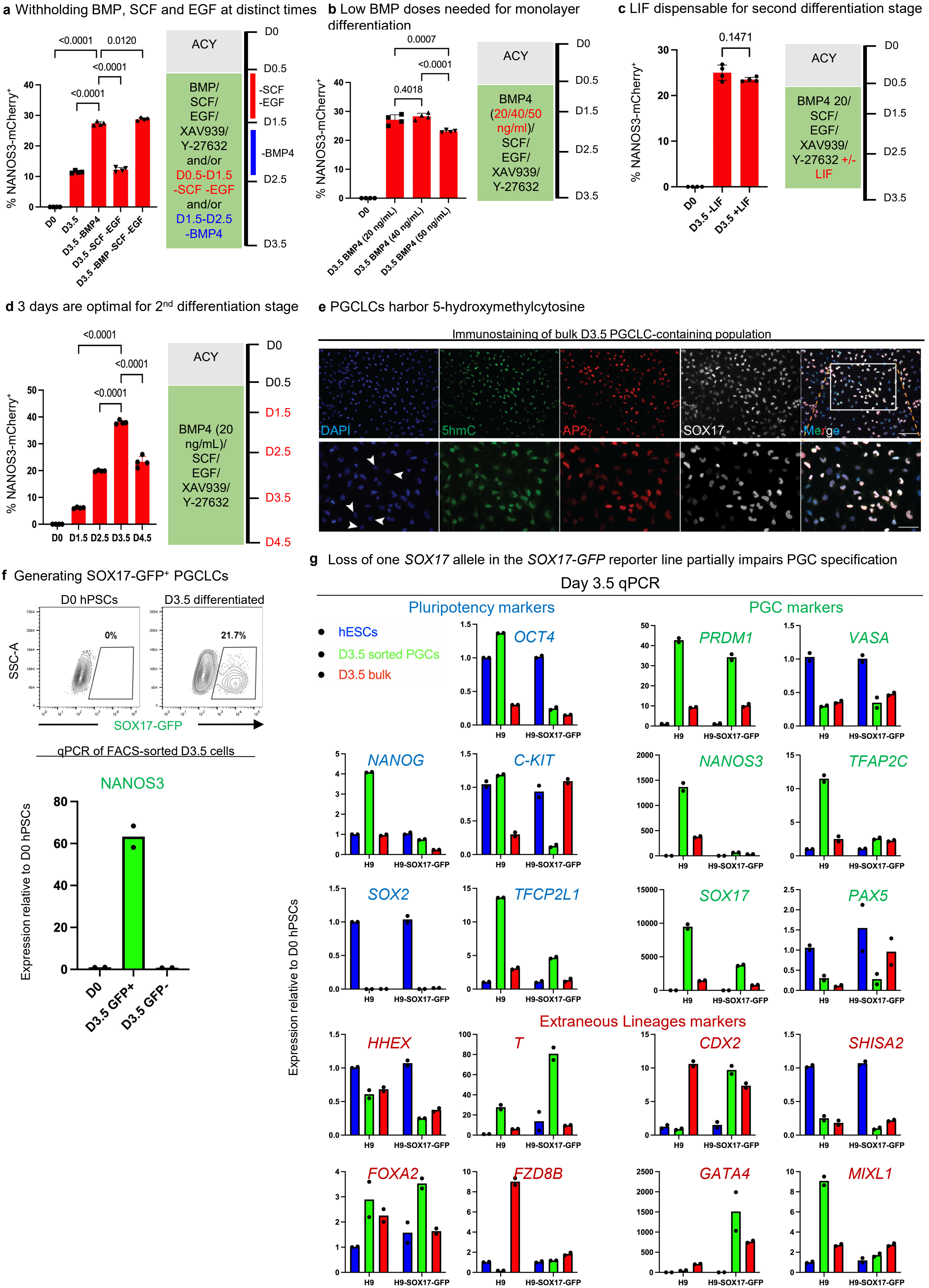
a Withholding BMP, SCF and EGF at distinct times
% NANOS3-mCherry⁺
D0
D3.5
D3.5 -BMP4
D3.5 -SCF -EGF
D3.5 -BMP -SCF -EGF
<0.0001
0.0120
ACY
BMP/ SCF/ EGF/ XAV939/ Y-27632 and/or
D0.5-D1.5 -SCF -EGF
and/or
D1.5-D2.5 -BMP4
-SCF -EGF
-BMP4
D0.5
D1.5
D2.5
b Low BMP doses needed for monolayer differentiation
0.0007
0.4018
D3.5 BMP4 (20 ng/mL)
D3.5 BMP4 (40 ng/mL)
D3.5 BMP4 (50 ng/mL)
BMP4 (20/40/50 ng/ml)/ SCF/ EGF/ XAV939/ Y-27632
c LIF dispensable for second differentiation stage
0.1471
D3.5 -LIF
D3.5 +LIF
BMP4 20/ SCF/ EGF/ XAV939/ Y-27632 +/- LIF
d 3 days are optimal for 2nd differentiation stage
D1.5
D2.5
D4.5
BMP4 (20 ng/mL)/ SCF/ EGF/ XAV939/ Y-27632
e PGCLCs harbor 5-hydroxymethylcytosine
Immunostaining of bulk D3.5 PGCLC-containing population
DAPI
5hmC
AP2γ
SOX17
Merge
f Generating SOX17-GFP⁺ PGCLCs
D0 hPSCs
D3.5 differentiated
SSC-A
0%
21.7%
SOX17-GFP
qPCR of FACS-sorted D3.5 cells
NANOS3
Expression relative to D0 hPSCs
D3.5 GFP+
D3.5 GFP-
g Loss of one SOX17 allele in the SOX17-GFP reporter line partially impairs PGC specification
Day 3.5 qPCR
Pluripotency markers
PGC markers
hESCs
D3.5 sorted PGCs
D3.5 bulk
OCT4
NANOG
C-KIT
SOX2
TFCP2L1
PRDM1
VASA
NANOS3
TFAP2C
SOX17
PAX5
Extraneous Lineages markers
HHEX
T
CDX2
SHISA2
FOXA2
FZD8B
GATA4
MIXL1
H9
H9-SOX17-GFP

Figure S4. Optimization of human PGCLC differentiation protocol in simplified monolayer conditions, related to Fig. 4

a. *NANOS3-mCherry* hPSCs were first differentiated into posterior epiblast (ACY = Activin + CHIR + Y27632 for 12 hrs); subsequently, for posterior epiblast differentiation into PGCs in the context of BMP4 + SCF + EGF + XAV939 + Y-27632 from D0.5-3.5, BMP4 subtraction from D1.5-2.5 and SCF/EGF subtraction from D0.5-1.5 both enhance efficiency, as assayed by flow cytometry analysis for NANOS3-mCherry fluorescent reporter expression on D3.5. N=4 biological replicates. Statistical analysis, one way ANOVA with Tukey's multiple comparison test. Source data are provided as a Source Data file.

b. BMP4 can be titrated to 20 ng/mL on D0.5-3.5 without significant loss in efficiency, as assayed by flow cytometry analysis for NANOS3-mCherry fluorescent reporter expression on D3.5. N=4 biological replicates. Statistical analysis, one way ANOVA with Tukey's multiple comparison test. Source data are provided as a Source Data file.

c. Addition of LIF during D0.5-3.5 does not improve efficiency, as assayed by flow cytometry analysis for NANOS3-mCherry fluorescent reporter expression on D3.5. N=4 biological replicates. Statistical analysis, one way ANOVA with Tukey's multiple comparison test. Source data are provided as a Source Data file.

d. In our hPSC-to-PGC differentiation protocol, mCherry expression peaks on D3.5 of differentiation, as assayed by flow cytometry analysis for NANOS3-mCherry fluorescent reporter expression on various timepoints. N=4 biological replicates. Statistical analysis, one way ANOVA with Tukey's multiple comparison test. Source data are provided as a Source Data file.

e. Representative images of H9 hESCs fixed at D3.5 and stained for the reported markers. Arrowheads indicate cells with decreased 5hmC signal, corresponding to non-hPGCLCs as highlighted by lack of expression of PGC specific markers from 3 independent experiments. Bottom row the subset of cells in the rectangle at higher magnification. Scale bar = 100um for top row, 50um for bottom row. DAPI was used as nuclear counterstain.

f. H9 *SOX17-GFP* hESCs were differentiated into PGCs and flow cytometry was performed to assay reporter expression before or after 3.5 days of differentiation (*top*); to confirm that SOX17-GFP^+ D3.5 cells are PGCs, qPCR was performed on D3.5 SOX17-GFP^+ and SOX17-$GFP^–$ cells for PGC marker *NANOS3*; as a negative control, undifferentiated hPSCs (D0) were also analyzed, and gene expression is shown relative to undifferentiated hPSCs (which was set = 1.0). N=2. Source data are provided as a Source Data file.

g. Comparison of wild-type H9 hESCs vs. *SOX17-GFP* hESCs (where one *SOX17* allele is replaced with *GFP*, thus generating functionally *SOX17*-heterozygous cells (Wang et al., 2011)) differentiated into PGCs; qPCR was performed on undifferentiated hESCs (D0), D3.5 bulk populations and D3.5 FACS-purified PGCs in the 2 genetic backgrounds; gene expression is shown relative to undifferentiated hPSCs (which was set = 1.0). N=2. Source data are provided as a Source Data file.

Fig. S5

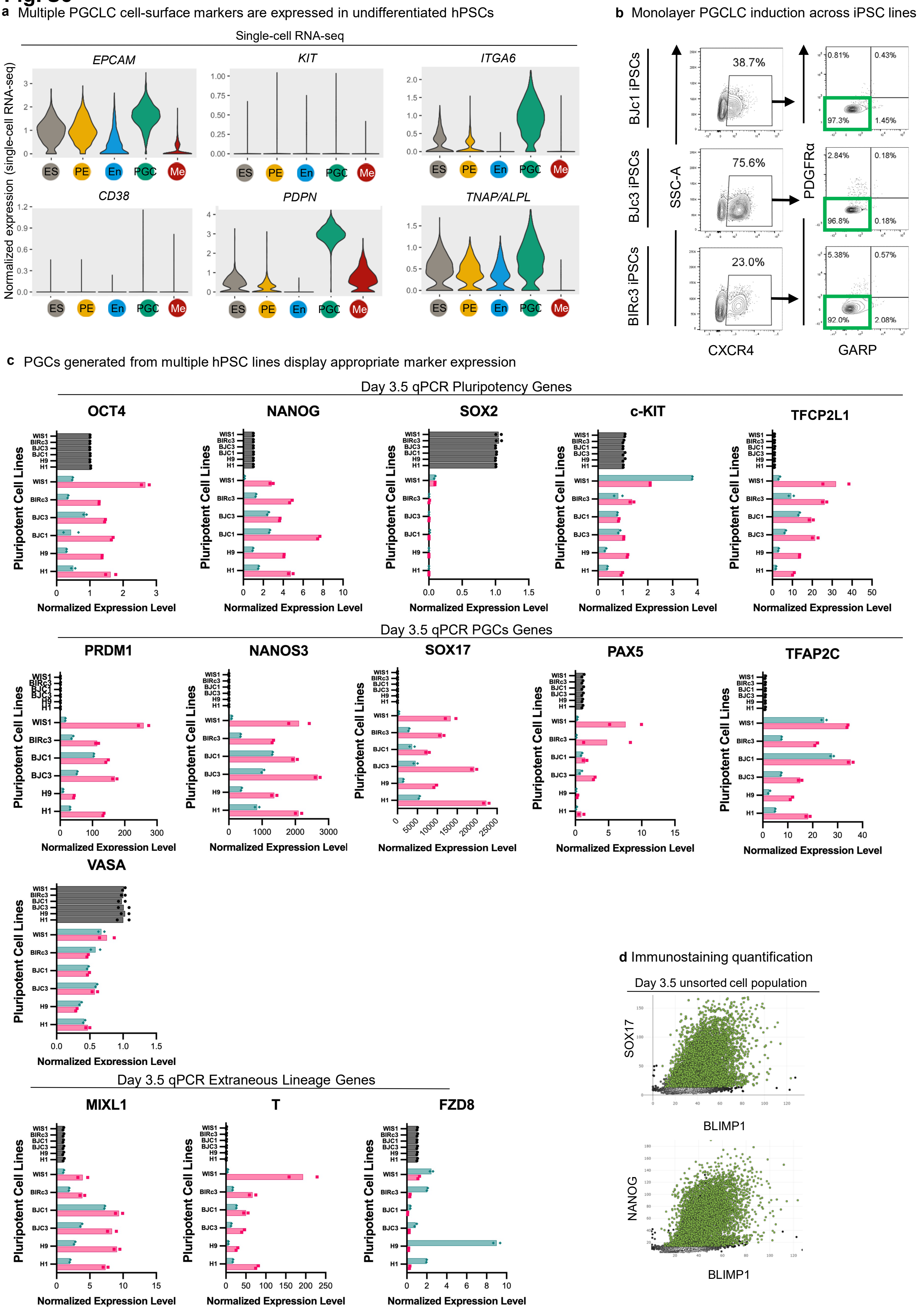
a Multiple PGCLC cell-surface markers are expressed in undifferentiated hPSCs
Single-cell RNA-seq
EPCAM
KIT
ITGA6
CD38
PDPN
TNAP/ALPL
Normalized expression (single-cell RNA-seq)
ES
PE
En
PGC
Me
b Monolayer PGCLC induction across iPSC lines
BJc1 iPSCs
BJc3 iPSCs
BIRc3 iPSCs
SSC-A
PDGFRα
CXCR4
GARP
38.7%
75.6%
23.0%
0.81%
0.43%
97.3%
1.45%
2.84%
0.18%
96.8%
0.18%
5.38%
0.57%
92.0%
2.08%
c PGCs generated from multiple hPSC lines display appropriate marker expression
Day 3.5 qPCR Pluripotency Genes
OCT4
NANOG
SOX2
c-KIT
TFCP2L1
Day 3.5 qPCR PGCs Genes
PRDM1
NANOS3
SOX17
PAX5
TFAP2C
VASA
Day 3.5 qPCR Extraneous Lineage Genes
MIXL1
T
FZD8
Pluripotent Cell Lines
Normalized Expression Level
WIS1
BIRc3
BJC1
BJC3
H9
H1
d Immunostaining quantification
Day 3.5 unsorted cell population
SOX17
NANOG
BLIMP1

Figure S5. Expression of surface markers in single cell RNA-seq data, related to Fig 5

a. Single-cell RNA-seq reveals the expression of transcripts that encode previously-reported cell-surface markers of PGCLCs (cell-types were classified as shown in

b. The indicated iPSC lines were used to induce hPGCLCs with the current protocol. Source data are provided as a Source Data file.

c. Gene expression of pluripotency, PGC-specific, and exogenous markers assesed by qPCR analysis of bulk D3.5 population (unsorted) and FACS-purified hPGCLCs. FACS analysis was performed based on the CXCR4+/PDGFRa-/GARP- surface signature. qPCR data were normalized to expression in undifferentiated cells (set at 1.0). N=2. Source data are provided as a Source Data file.

d. Representative fluorescence threshold determination for immunofluorescence quantification of D3.5 unsorted cells based on the 3 reported PGC-markers. See Materials and Methods for analysis details.

Fig. S6

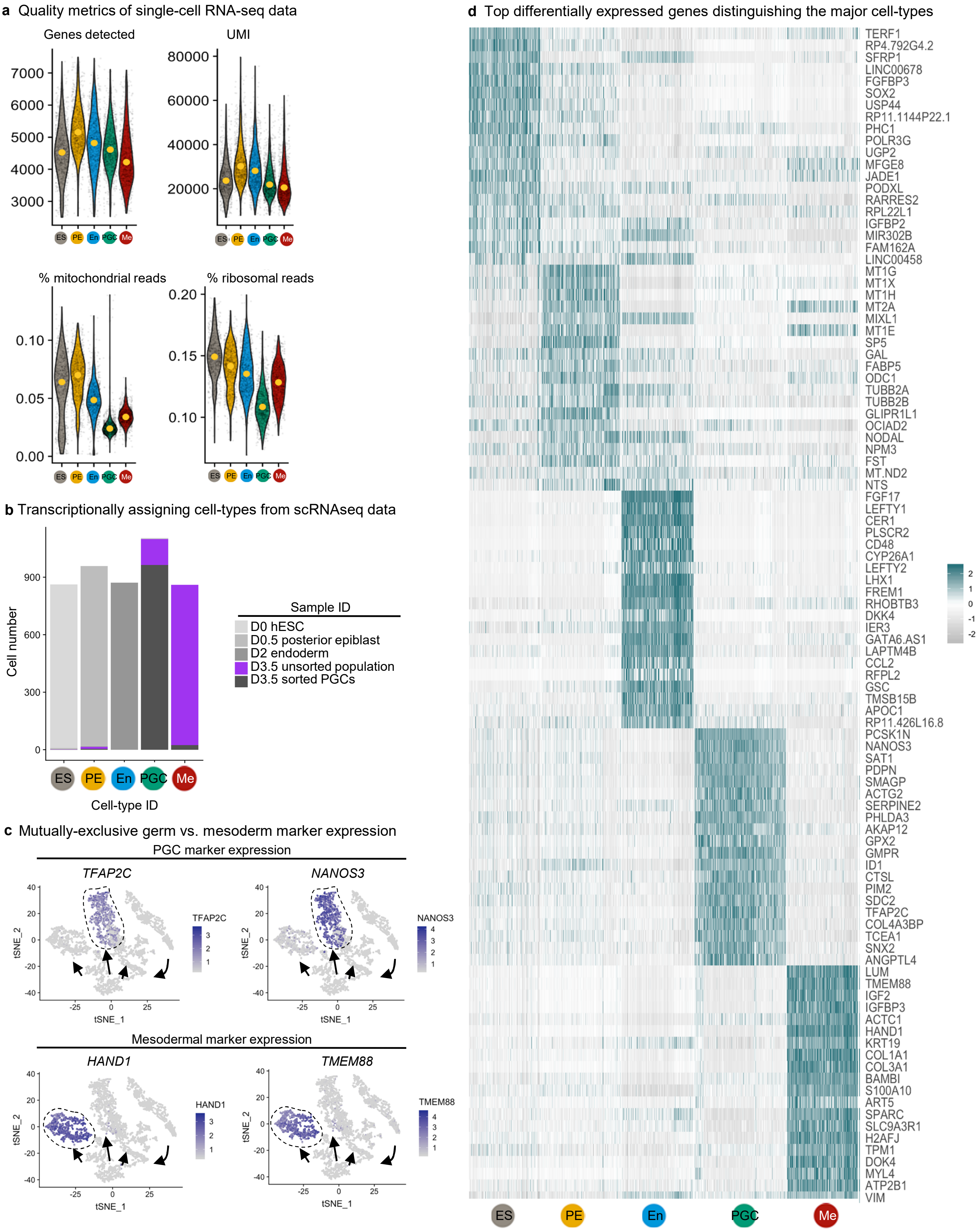
a Quality metrics of single-cell RNA-seq data
Genes detected
7000
6000
5000
4000
3000
UMI
80000
60000
40000
20000
ES PE En PGC Me
% mitochondrial reads
0.10
0.05
0.00
% ribosomal reads
0.20
0.15
0.10
b Transcriptionally assigning cell-types from scRNAseq data
Cell number
900
600
300
0
Sample ID
D0 hESC
D0.5 posterior epiblast
D2 endoderm
D3.5 unsorted population
D3.5 sorted PGCs
ES PE En PGC Me
Cell-type ID
c Mutually-exclusive germ vs. mesoderm marker expression
PGC marker expression
TFAP2C
NANOS3
Mesodermal marker expression
HAND1
TMEM88
tSNE_1
tSNE_2
40
20
0
-20
-40
-25
0
25
4
3
2
1
d Top differentially expressed genes distinguishing the major cell-types
TERF1
RP4.792G4.2
SFRP1
LINC00678
FGFBP3
SOX2
USP44
RP11.1144P22.1
PHC1
POLR3G
UGP2
MFGE8
JADE1
PODXL
RARRES2
RPL22L1
IGFBP2
MIR302B
FAM162A
LINC00458
MT1G
MT1X
MT1H
MT2A
MIXL1
MT1E
SP5
GAL
FABP5
ODC1
TUBB2A
TUBB2B
GLIPR1L1
OCIAD2
NODAL
NPM3
FST
MT.ND2
NTS
FGF17
LEFTY1
CER1
PLSCR2
CD48
CYP26A1
LEFTY2
LHX1
FREM1
RHOBTB3
DKK4
IER3
GATA6.AS1
LAPTM4B
CCL2
RFPL2
GSC
TMSB15B
APOC1
RP11.426L16.8
PCSK1N
NANOS3
SAT1
PDPN
SMAGP
ACTG2
SERPINE2
PHLDA3
AKAP12
GPX2
GMPR
ID1
CTSL
PIM2
SDC2
TFAP2C
COL4A3BP
TCEA1
SNX2
ANGPTL4
LUM
TMEM88
IGF2
IGFBP3
ACTC1
HAND1
KRT19
COL1A1
COL3A1
BAMBI
S100A10
ART5
SPARC
SLC9A3R1
H2AFJ
TPM1
DOK4
MYL4
ATP2B1
VIM
2
1
0
-1
-2
ES PE En PGC Me

Figure S6. Quality control and cell-type-specific, differentially expressed genes from single-cell RNA-seq datasets, related to Fig. 7

a. Violin plot of genes detected, unique molecular identifier (UMI) counts, percent mitochondrial reads and percent ribosomal reads across all 5 cell-type clusters (ES = undifferentiated hESCs; PE = posterior epiblast; En = endoderm; Me = mesoderm [non-PGC])
b. Proportion of cells from different libraries in each cluster; N.B. PGCs are found in both the D3.5 bulk as well as the D3.5 FACS-sorted PGC samples, whereas mesoderm cells are found almost exclusively in the D3.5 bulk population but not the D3.5 FACS-sorted PGC samples
c. *t*-SNE plot with overlaid expression of PGC and mesoderm markers showing that these markers are expressed in a mutually-exclusive fashion in different cell clusters
d. Heatmap of top differentially expressed genes across the 5 major clusters (i.e., cell-types)

Fig. S7

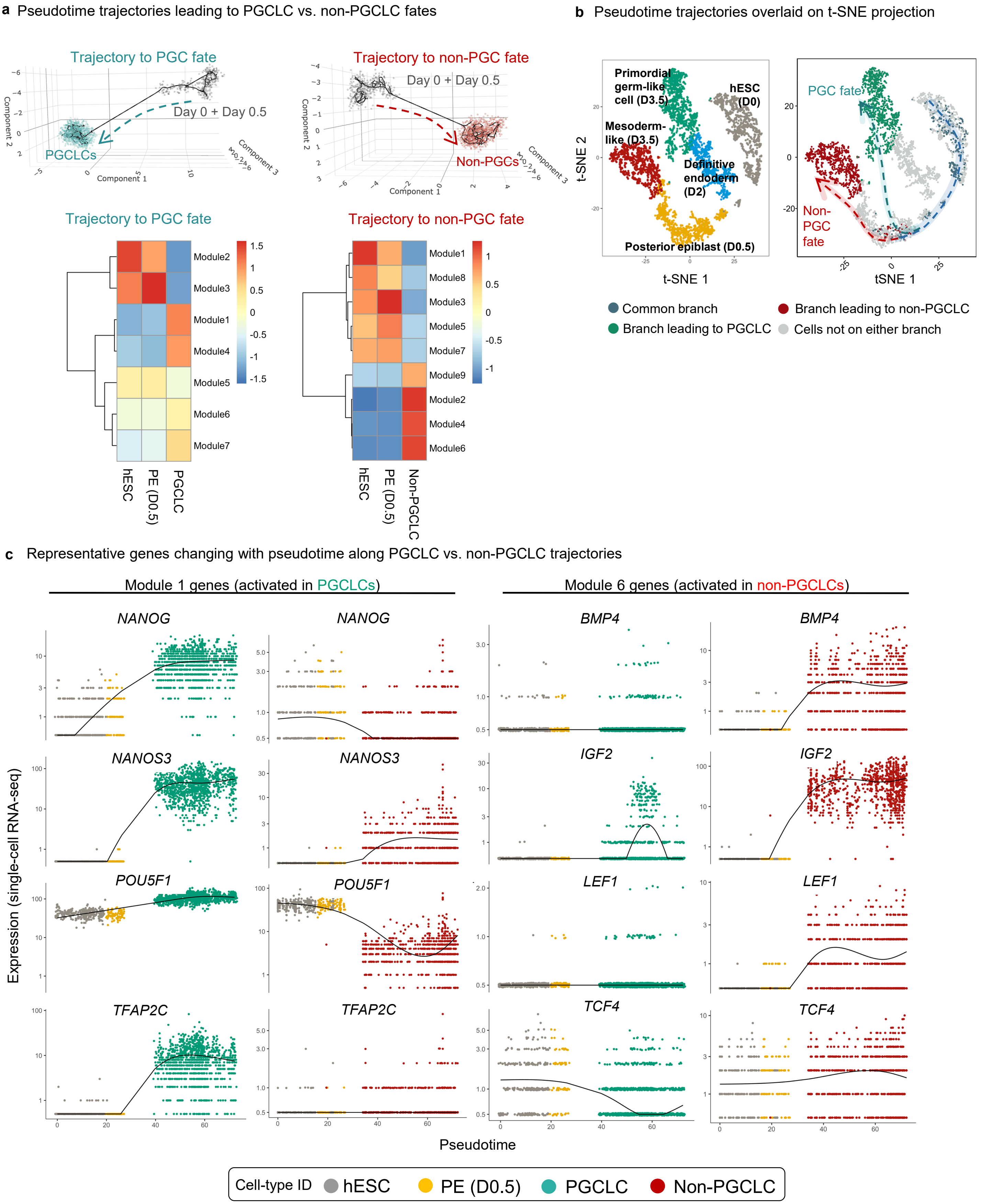
a Pseudotime trajectories leading to PGCLC vs. non-PGCLC fates
b Pseudotime trajectories overlaid on t-SNE projection
Trajectory to PGC fate
Day 0 + Day 0.5
PGCLCs
Component 1
Component 2
Component 3
Trajectory to non-PGC fate
Non-PGCs
Module1
Module2
Module3
Module4
Module5
Module6
Module7
Module8
Module9
hESC
PE (D0.5)
PGCLC
Non-PGCLC
Primordial germ-like cell (D3.5)
hESC (D0)
Mesoderm-like (D3.5)
Definitive endoderm (D2)
Posterior epiblast (D0.5)
t-SNE 1
t-SNE 2
PGC fate
Non-PGC fate
tSNE 1
Common branch
Branch leading to PGCLC
Branch leading to non-PGCLC
Cells not on either branch
c Representative genes changing with pseudotime along PGCLC vs. non-PGCLC trajectories
Module 1 genes (activated in PGCLCs)
Module 6 genes (activated in non-PGCLCs)
NANOG
NANOS3
POU5F1
TFAP2C
BMP4
IGF2
LEF1
TCF4
Expression (single-cell RNA-seq)
Pseudotime
Cell-type ID
hESC
PE (D0.5)
PGCLC
Non-PGCLC

Figure S7. Trajectory analysis of hPSC differentiation to PGCLCs, related to Fig. 7

a. Pseudotemporal ordering of hESCs differentiating to PGCLCs, showing branchpoint analysis, leading to PGCLC fate vs. non-PGCLC (mesoderm-like cells) fate (top). Modules of genes significantly changing with pseudotime on trajectory to PGCLC fate vs. non-PGCLC fate (bottom).
b. Cells from PGCLC trajectory vs. non-PGCLC trajectory (from A) overlaid on tSNE analysis.
c. Representative genes from Module 1 on trajectory to PGCLC fate and Module 6 on trajectory to non-PGCLC fate.

Fig. S8

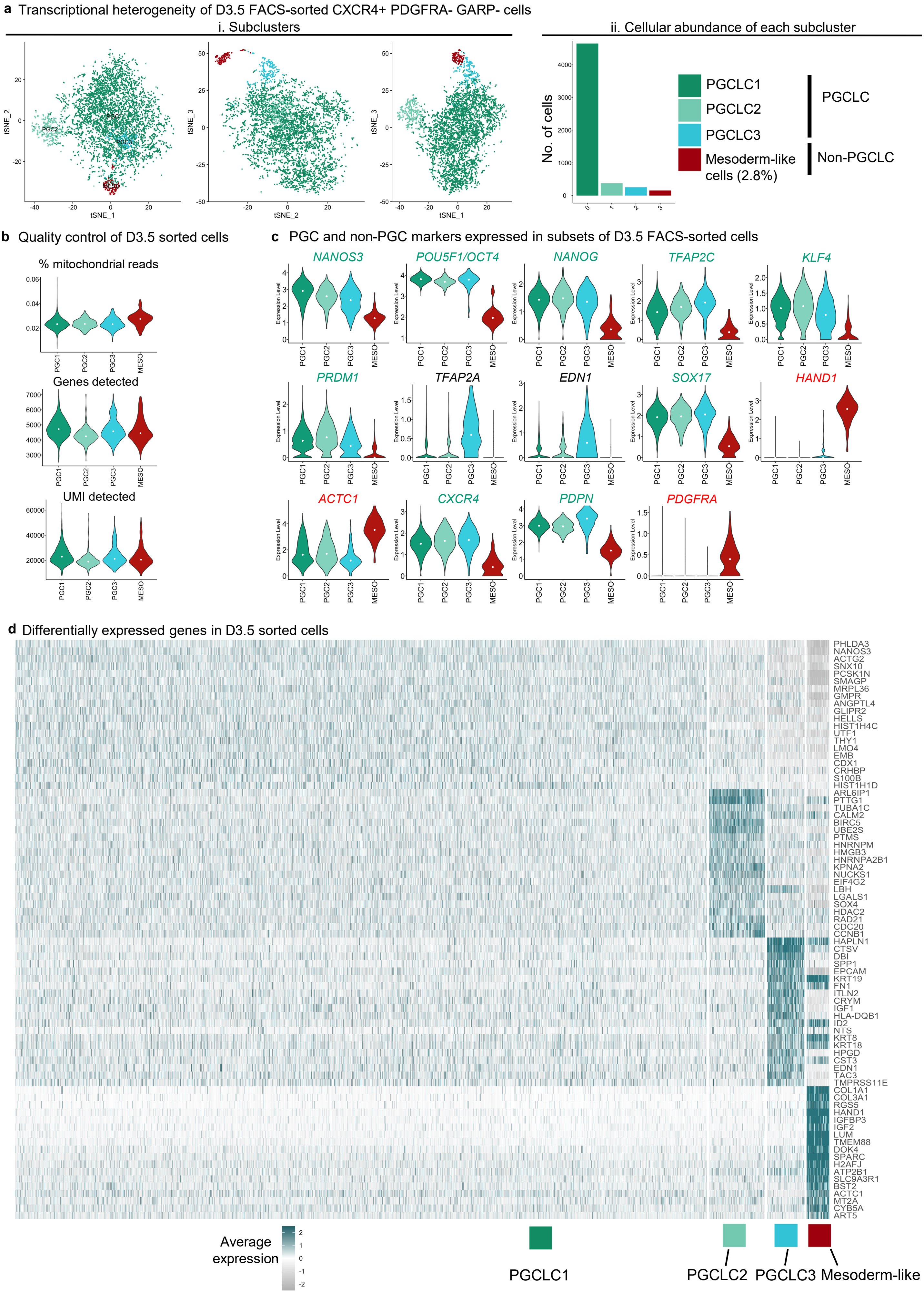
a Transcriptional heterogeneity of D3.5 FACS-sorted CXCR4+ PDGFRA- GARP- cells
i. Subclusters
ii. Cellular abundance of each subcluster
tSNE_1
tSNE_2
tSNE_3
PGC1
PGC2
PGC3
No. of cells
PGCLC1
PGCLC2
PGCLC3
Mesoderm-like cells (2.8%)
PGCLC
Non-PGCLC
b Quality control of D3.5 sorted cells
% mitochondrial reads
Genes detected
UMI detected
c PGC and non-PGC markers expressed in subsets of D3.5 FACS-sorted cells
NANOS3
POU5F1/OCT4
NANOG
TFAP2C
KLF4
PRDM1
TFAP2A
EDN1
SOX17
HAND1
ACTC1
CXCR4
PDPN
PDGFRA
Expression Level
PGC1
PGC2
PGC3
MESO
d Differentially expressed genes in D3.5 sorted cells
PHLDA3
NANOS3
ACTG2
SNX10
PCSK1N
SMAGP
MRPL36
GMPR
ANGPTL4
GLIPR2
HELLS
HIST1H4C
UTF1
THY1
LMO4
EMB
CDX1
CRHBP
S100B
HIST1H1D
ARL6IP1
PTTG1
TUBA1C
CALM2
BIRC5
UBE2S
PTMS
HNRNPM
HMGB3
HNRNPA2B1
KPNA2
NUCKS1
EIF4G2
LBH
LGALS1
SOX4
HDAC2
RAD21
CDC20
CCNB1
HAPLN1
CTSV
DBI
SPP1
EPCAM
KRT19
FN1
ITLN2
CRYM
IGF1
HLA-DQB1
ID2
NTS
KRT8
KRT18
HPGD
CST3
EDN1
TAC3
TMPRSS11E
COL1A1
COL3A1
RGS5
HAND1
IGFBP3
IGF2
LUM
TMEM88
DOK4
SPARC
H2AFJ
ATP2B1
SLC9A3R1
BST2
ACTC1
MT2A
CYB5A
ART5
Average expression
PGCLC1
PGCLC2
PGCLC3
Mesoderm-like

Figure S8: Heterogeneity within Day 3.5 sorted PGCLCs, related to Fig.7.

a. Clustering analysis of Day 3.5 sorted PGCLCs, showing 4 subclusters (i). Number of cells per cluster in Day 3.5 sorted PGCLCs. 2.8% of cells were mesoderm-like, non-PGCLCs (ii).
b. Quality control analysis of Day 3.5 sorted PGCLCs, showing percent mitochondrial genes detected, total number of genes detected per cluster, and total unique molecular identifiers (UMI) detected per cluster.
c. Violin plot showing expression levels of PGC and non-PGC markers across clusters.
d. Heatmap of differentially expressed genes across clusters.

Fig. S9

a Bifurcation of NANOG⁺ hPSCs/posterior epiblast into NANOG⁺ CXCR4⁺ PGCs vs. NANOG⁻ CXCR4⁻ non-PGCs

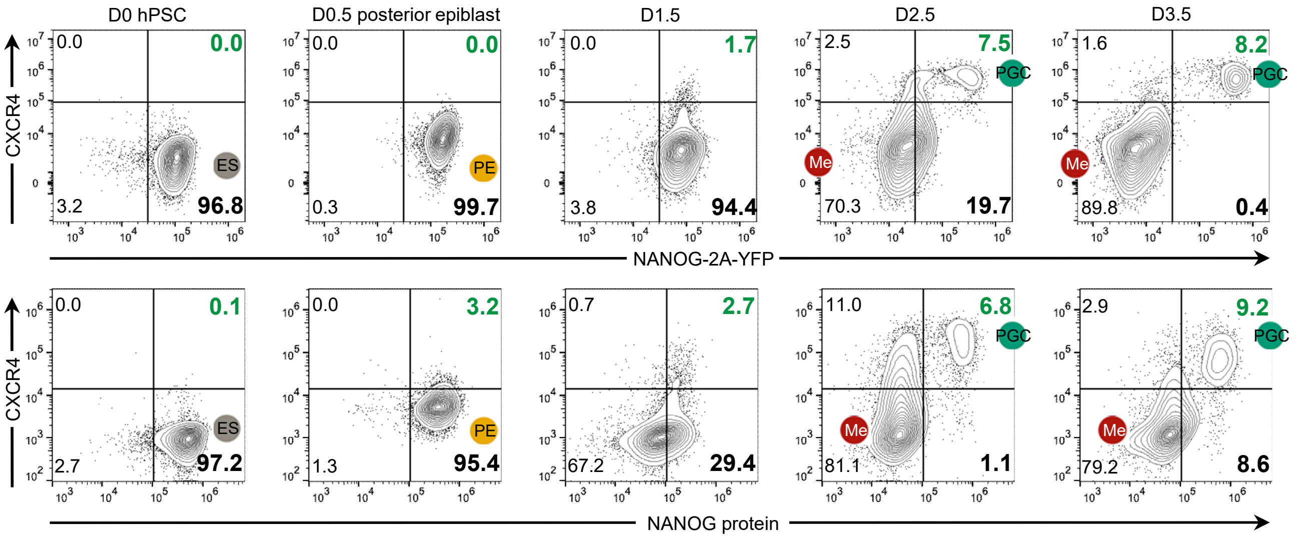

b NANOG protein continuously expressed during PGCLC formation

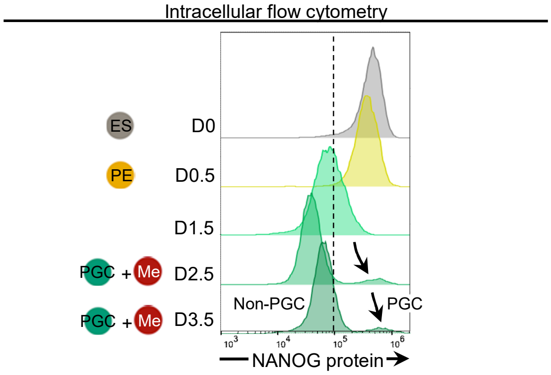

c SOX17 becomes co-expressed with OCT4 and NANOG

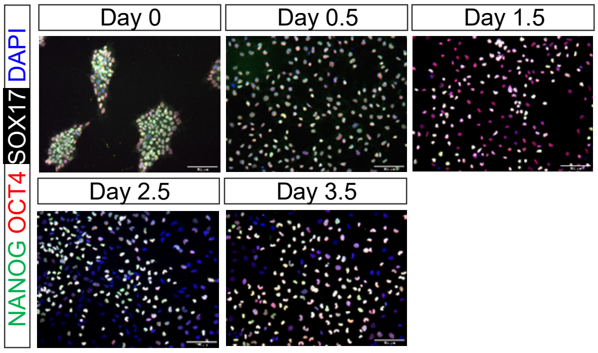

d qPCR quantification of *NANOG* siRNA knockdown

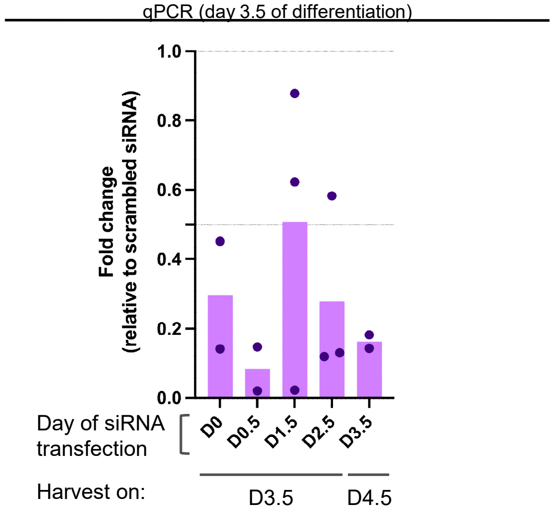

Figure S9: Continuous pluripotency factor expression during PGCLC differentiation, related to Fig. 8.

a. Flow cytometry analysis of *NANOG-2A-YFP* hPSCs differentiated towards PGCs, with surface staining of CXCR4 combined with either flow cytometry analysis of NANOG-2A-YFP reporter activity or else intracellular staining of NANOG protein itself. Source data are provided as a Source Data file.

b. Intracellular flow cytometry for NANOG protein expression during hPSC differentiation towards PGCs.

c. Representative merged immunofluorescence images of H9 stained at each timepoint during the 3.5 days of differentiation with the indicated markers, from 3 independent experiments. Scale bar = 100mm.

d. NANOG expression in unsorted siRNA (targeting NANOG) transfected cells relative to cells transfected with scrambled siRNA measured by qPCR. Days on which siRNA transfection was started and when they were harvest is shown below. N=2 biological replicates/group for all except Day 1.5 and Day 2.5, and n=3 biological replicates/group.

Fig. S10

a Computationally downsampling D3.5 PGCLCs analyzed by single-cell RNA-seq

Number of D3.5 cells	300	545	1362	2724	4085	5447
Downsampling (%)	94%	90%	75%	50%	25%	0%
Genes detected	16,590	17,190	17,617	17,643	17,645	17,645
Genes detected (%)	94%	97%	100%	100%	100%	100%

b Single-cell RNA-seq of *in vivo* fetal germ and somatic cells and *in vitro* PGCLCs

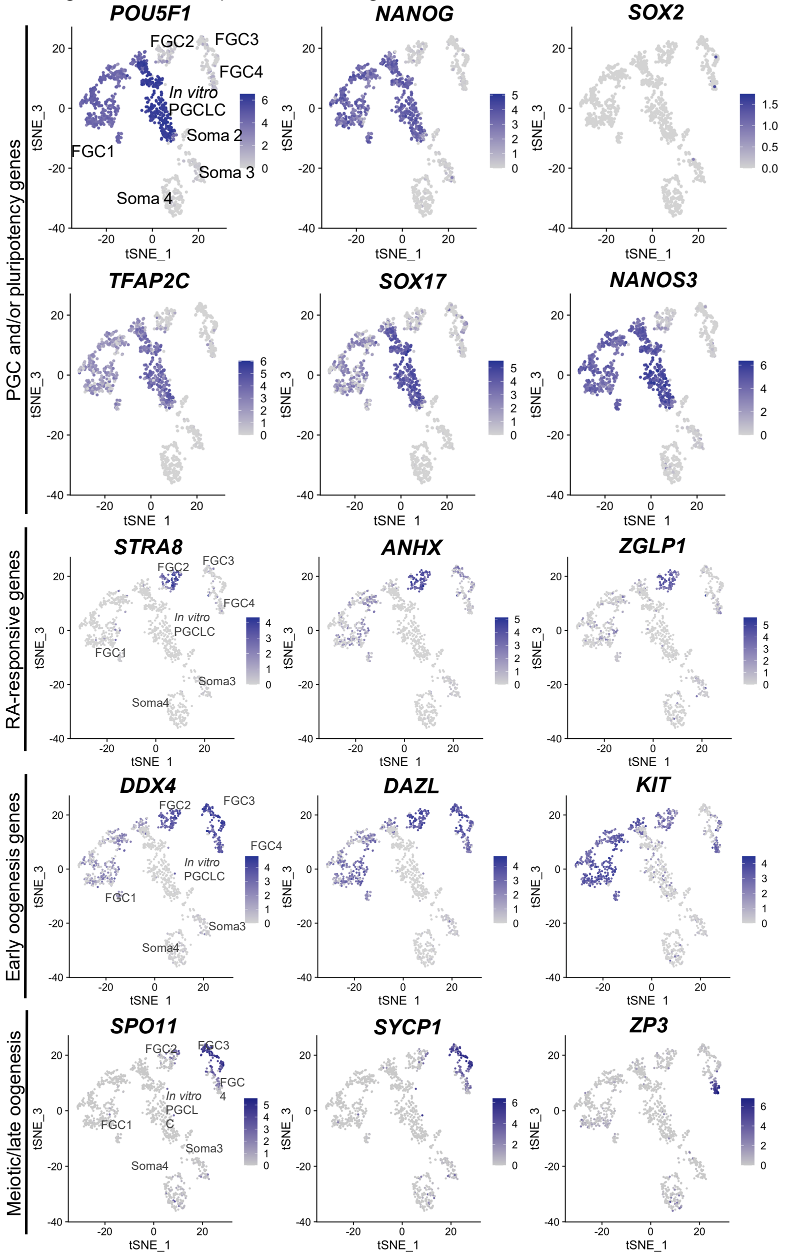

c Clustering of *in vivo* fetal PGCs, somatic cells and *in vitro* PGCLCs

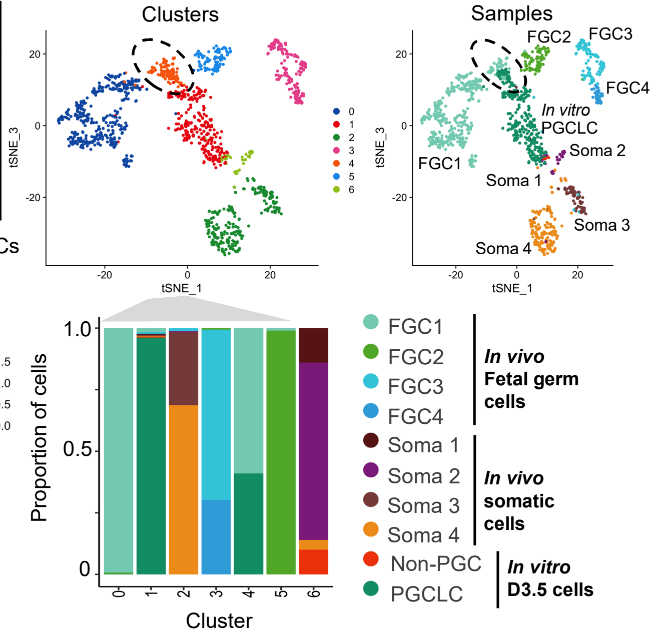

d Gene ontology of differentially expressed genes between clusters

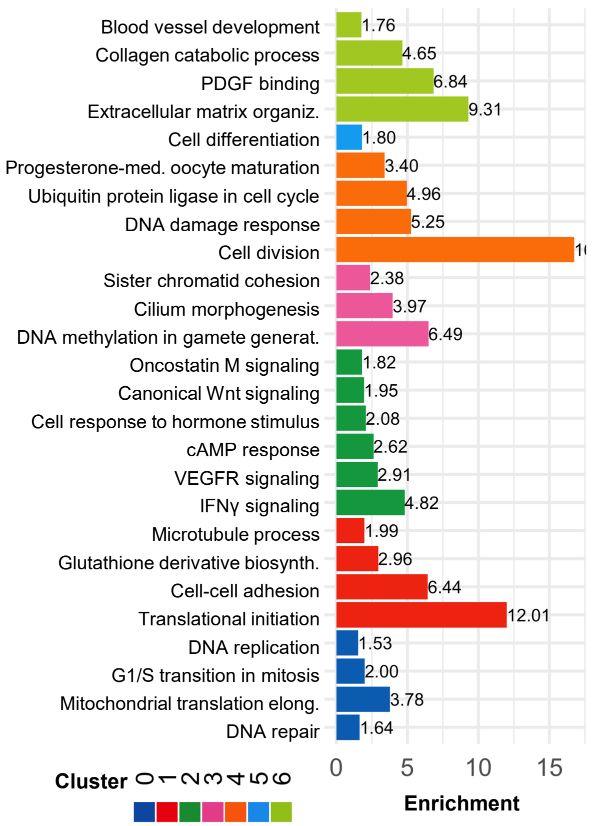

e Top 10 differentially expressed genes between clusters

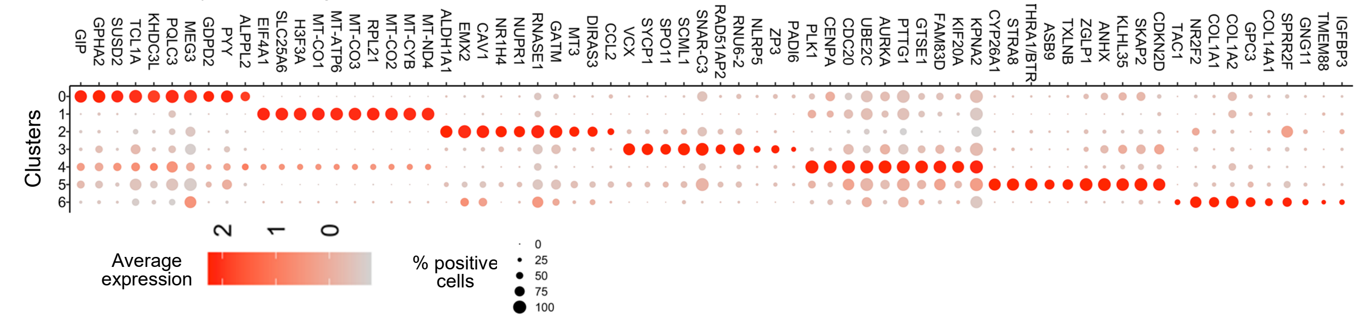

Figure S10: Single-cell RNA-seq comparison of hPSC-derived PGCLCs *in vitro* vs. human fetal germ cells *in vivo*, related to Fig. 9.

a. In the scRNA-seq comparisons of hPSC-derived PGCLCs *in vitro* vs. human fetal germ cells and somatic cells *in vivo*, we randomly selected a subset of PGCLCs ("downsampling") for further analysis (**Supplementary Methods**). hPSC-derived FACS-sorted $CXCR4^{+}$ $GARP^{-}$ $PDGFRA^{-}$ D3.5 PGCLCs were used for this analysis, and we progressively downsampled the population and assessed how many genes across the transcriptome were expressed in at least one cell.
b. Expression patterns of early PGC markers, RA-responsive genes, early oogenesis markers and meiotic/late oogenesis genes, depicted on *t*-SNE plots of *in vivo*-derived FGCs and somatic cells (Li et al., 2017) and *in vitro*-derived FACS-sorted $CXCR4^{+}$ $PDGFRA^{-}$ $GARP^{-}$ D3.5 PGCLCs.
c. Integrated clustering of human fetal female germ cells clusters (FGC1-4) and somatic cells clusters (Soma1-4) with *in vitro* Day 3.5 sorted PGCLCs and non-PGCLCs. tSNE clustering analysis show 6 different clusters (*left*) and samples color coded (*right*). Below, the fraction of cells from each sample within each cluster is reported.
d. Gene ontology enrichment analysis of biological processes enriched in the different clusters.
e. Top 10 differentially expressed genes between clusters.

Fig. S11

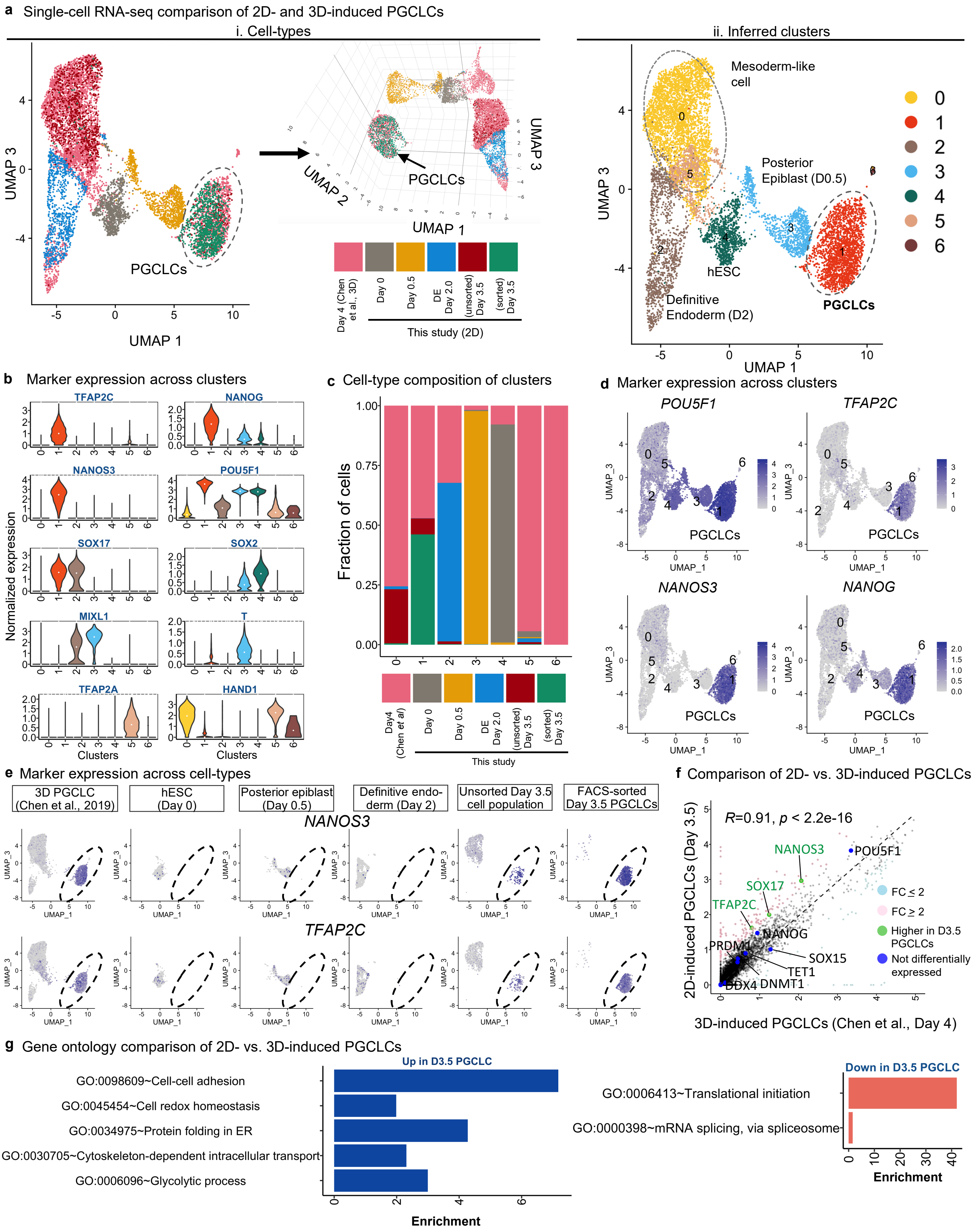
a Single-cell RNA-seq comparison of 2D- and 3D-induced PGCLCs
i. Cell-types
ii. Inferred clusters
UMAP 1
UMAP 2
UMAP 3
PGCLCs
Day 4 (Chen et al., 3D)
Day 0
Day 0.5
DE Day 2.0
(unsorted) Day 3.5
(sorted) Day 3.5
This study (2D)
Mesoderm-like cell
Posterior Epiblast (D0.5)
hESC
Definitive Endoderm (D2)
PGCLCs
0
1
2
3
4
5
6
b Marker expression across clusters
TFAP2C
NANOG
NANOS3
POU5F1
SOX17
SOX2
MIXL1
T
TFAP2A
HAND1
Normalized expression
Clusters
c Cell-type composition of clusters
Fraction of cells
Day4 (Chen et al)
Day 0
Day 0.5
DE Day 2.0
(unsorted) Day 3.5
(sorted) Day 3.5
This study
d Marker expression across clusters
POU5F1
TFAP2C
NANOS3
NANOG
PGCLCs
UMAP_1
UMAP_3
e Marker expression across cell-types
3D PGCLC (Chen et al., 2019)
hESC (Day 0)
Posterior epiblast (Day 0.5)
Definitive endoderm (Day 2)
Unsorted Day 3.5 cell population
FACS-sorted Day 3.5 PGCLCs
NANOS3
TFAP2C
f Comparison of 2D- vs. 3D-induced PGCLCs
R=0.91, p < 2.2e-16
NANOS3
POU5F1
SOX17
TFAP2C
NANOG
PRDM1
SOX15
TET1
DDX4
DNMT1
FC ≤ 2
FC ≥ 2
Higher in D3.5 PGCLCs
Not differentially expressed
2D-induced PGCLCs (Day 3.5)
3D-induced PGCLCs (Chen et al., Day 4)
g Gene ontology comparison of 2D- vs. 3D-induced PGCLCs
Up in D3.5 PGCLC
GO:0098609~Cell-cell adhesion
GO:0045454~Cell redox homeostasis
GO:0034975~Protein folding in ER
GO:0030705~Cytoskeleton-dependent intracellular transport
GO:0006096~Glycolytic process
Enrichment
Down in D3.5 PGCLC
GO:0006413~Translational initiation
GO:0000398~mRNA splicing, via spliceosome
Enrichment

Figure S11: Comparison of PGCLCs from this study with *in vitro* derived human PGCLCs from Chen *et al,* 2019.

a. Integrated clustering of Day 4 PGCLCs from Chen *et al*, 2019, Cell Reports (down sampled to 5000 cells) with cells from this study (down sampled to 1000 cells per group) (i). Clustering analysis of integrated data in (A) (ii).
b. Violin plot of expression levels of key PGC markers across clusters.
c. Fraction of cells from each group present within each cluster.
d. Expression of key PGC markers shown across clusters.
e. Expression of key PGC markers within each cell group.
f. Scatter plot showing correlation between Day 3.5 sorted PGCLCs (this study) and Day 4 PGCLCs (Chen *et al*, 2019, Cell Reports). Key PGC markers genes expressed at 2-fold higher level in Day3.5 sorted PGCLCs compared to Day4 PGCLCs (Chen *et al*, 2019, Cell Reports) are shown in green; key PGC marker genes that are not differentially expressed are shown in blue. Statistical test - Pearson correlation with 95% confidence interval, P-value < 2.2e-16.
g. Gene ontology enrichment analysis of biological processes in Day 3.5 PGCLCs and Day 4 PGCLCs from Chen *et al*, 2019.
