# Supplementary material for: Monolayer platform to generate and purify primordial germ-like cells in vitro provides insights into human germline specification
Source: Nat Commun. 2023 Sep 14;14:5690. doi: 10.1038/s41467-023-41302-w (PMC10502105; doi:10.1038/s41467-023-41302-w)
Supplement: Supplementary file 3 — Description of Additional Supplementary Files [file 41467_2023_41302_MOESM3_ESM.pdf]

Description of Additional Supplementary Files

File Name: Supplementary Data 1
Description: Combined hESC DE PGC Legendscreen Analysis

File Name: Supplementary Data 2
Description: Gene Modules Changing by pseudotime trajectory to PGC vs non PGC

File Name: Supplementary Data 3
Description: Expression of FGCs Soma PGCLCs

File Name: Supplementary Data 4
Description: Genes differentially expressed between d4 PGCLCs in cluster 1

File Name: Supplementary Data 5
Description: DEG Bulk RNASeq

File Name: Supplementary Data 6
Description: Reagents and Primers

File Name: Supplementary Movie 1
Description: Time Lapse of PGCLCs differentiation and NANOG-YFP expression in incipiently forming PGCLCs
