# Supplementary material for: Monolayer platform to generate and purify primordial germ-like cells in vitro provides insights into human germline specification
Source: Nat Commun. 2023 Sep 14;14:5690. doi: 10.1038/s41467-023-41302-w (PMC10502105; doi:10.1038/s41467-023-41302-w)
Supplement: Supplementary file 11 — Reporting Summary [file 41467_2023_41302_MOESM11_ESM.pdf]

nature research

Corresponding author(s): Dr. Vittorio Sebastiano

Last updated by author(s): Jul 11, 2023

Reporting Summary

Nature Research wishes to improve the reproducibility of the work that we publish. This form provides structure for consistency and transparency in reporting. For further information on Nature Research policies, see our Editorial Policies and the Editorial Policy Checklist.

Statistics

For all statistical analyses, confirm that the following items are present in the figure legend, table legend, main text, or Methods section.

n/a	Confirmed	
☐	☒	The exact sample size (*n*) for each experimental group/condition, given as a discrete number and unit of measurement
☐	☒	A statement on whether measurements were taken from distinct samples or whether the same sample was measured repeatedly
☐	☒	The statistical test(s) used AND whether they are one- or two-sided<br>*Only common tests should be described solely by name; describe more complex techniques in the Methods section.*
☒	☐	A description of all covariates tested
☒	☐	A description of any assumptions or corrections, such as tests of normality and adjustment for multiple comparisons
☒	☐	A full description of the statistical parameters including central tendency (e.g. means) or other basic estimates (e.g. regression coefficient) AND variation (e.g. standard deviation) or associated estimates of uncertainty (e.g. confidence intervals)
☒	☐	For null hypothesis testing, the test statistic (e.g. *F*, *t*, *r*) with confidence intervals, effect sizes, degrees of freedom and *P* value noted<br>*Give P values as exact values whenever suitable.*
☒	☐	For Bayesian analysis, information on the choice of priors and Markov chain Monte Carlo settings
☒	☐	For hierarchical and complex designs, identification of the appropriate level for tests and full reporting of outcomes
☒	☐	Estimates of effect sizes (e.g. Cohen's *d*, Pearson's *r*), indicating how they were calculated

Our web collection on statistics for biologists contains articles on many of the points above.

Software and code

Policy information about availability of computer code

Data collection	BD FACSDiva was used for FACS data collection. Essen IncuCyte Zoom live cell imaging station (Essen BioScience, Ann Arbor, MI, USA) housed within a standard tissue culture incubator was used to perform live imaging analysis.<br>For comparative analysis of in vitro differentiate PGCLCs with in vivo human fetal PGCs, in vivo data was obtained from GSE86146.
Data analysis	FlowJo v10.6.2 was used for FACS data processing. GraphPad Prism 7 was used for figure generation.<br><br>Immunocytochemistry slides were imaged on a Leica DM4000 B equipped with a QImaging Retiga-2000R digital camera with a 20X or 40X objective, and processed using FIJI (v.1.52p).<br><br>Live imaging was performed using the Essen IncuCyte Zoom live cell imaging station (Essen BioScience, Ann Arbor, MI, USA) housed within a standard tissue culture incubator. Images were recorder every 30 minutes at 20X magnification both for phase and fluorescence imaging. Raw data were background corrected using the proprietary IncuCyte ZOOM Live-Cell Analysis System software and then analyzed with Fiji (v.1.52p).<br><br>Published software: a combination of NGS analysis tools were used for single-cell RNA-sequencing data analysis:<br>Cell Ranger v2.0 - processing of raw 10x single cell data, alignment and barcode UMI counting.<br>Seurat R package v2.3.1, v2.3.4, v3.0 and v4.0.1, R bioconductor (ggplot2, GGally), Monocle R package v2.0, Monocle 3 v1.0.0, DESeq2 (1.34.0), pheatmap (1.0.12), EnhancedVolcano (1.12.0), R stats package (4.1.2), and plotly (4.10.0).<br>Bulk RNA seq mapping - skewer (v0.2.2) and Salmon (v1.7.0).

For manuscripts utilizing custom algorithms or software that are central to the research but not yet described in published literature, software must be made available to editors and reviewers. We strongly encourage code deposition in a community repository (e.g. GitHub). See the Nature Research guidelines for submitting code & software for further information.

Data

Policy information about availability of data

All manuscripts must include a data availability statement. This statement should provide the following information, where applicable:

- Accession codes, unique identifiers, or web links for publicly available datasets
- A list of figures that have associated raw data
- A description of any restrictions on data availability

All sequencing data are under the super series GSE210712 - All single cell RNA-seq data counts matrix have been deposited in GEO database - GSE157475. All bulk RNA seq data have been uploaded under -- GSE210712.

Field-specific reporting

Please select the one below that is the best fit for your research. If you are not sure, read the appropriate sections before making your selection.

☒ Life sciences ☐ Behavioural & social sciences ☐ Ecological, evolutionary & environmental sciences

For a reference copy of the document with all sections, see nature.com/documents/nr-reporting-summary-flat.pdf

Life sciences study design

All studies must disclose on these points even when the disclosure is negative.

Sample size	No formal sample size calculation was performed for in vitro differentiated hPSCs that underwent single cell RNA-sequencing.<br>For all FACS and qPCR experiments, each condition consisted of two biological replicates each split into two technical replicates, for a total of 4 replicates per condition.
Data exclusions	Cells that were excluded via standard FACS gating strategies (see below) were not included in downstream analyses.
Replication	Fig. 3 outlines the reproducibility of our experimental approach across 6 additional cell types including 3 hESC and 3 iPSC lines.
Randomization	For single cell data anlaysis, randomized down-sampling was performed for combined analysis of H9 (Day0), D0.5, D3.5 sorted and D3.5 unsorted and DE analysis.
Blinding	Investigators were not blinded during group allocations. However, results have been verified by several independent investigators.

Reporting for specific materials, systems and methods

We require information from authors about some types of materials, experimental systems and methods used in many studies. Here, indicate whether each material, system or method listed is relevant to your study. If you are not sure if a list item applies to your research, read the appropriate section before selecting a response.

Materials & experimental systems

n/a	Involved in the study
☐	☒ Antibodies
☐	☒ Eukaryotic cell lines
☒	☐ Palaeontology and archaeology
☒	☐ Animals and other organisms
☒	☐ Human research participants
☒	☐ Clinical data
☒	☐ Dual use research of concern

Methods

n/a	Involved in the study
☒	☐ ChIP-seq
☐	☒ Flow cytometry
☒	☐ MRI-based neuroimaging

Antibodies

Antibodies used	Mouse anti-AP-2γ (6E4/4), Santa Cruz Biotechnologies, sc-12762, 1:50<br>Rat anti-Blimp1 (6D3), eBioscience, 14-5963-82, 1:50<br>Goat anti-Hand1, R&D systems, AF3168, 1:100<br>Rabbit anti-Nanog, Abcam, ab109250, 1:250<br>Mouse anti-Oct4, BD, 611203, 1:100<br>Goat anti-Sox17, R&D systems, AF1924, 1:500<br>APC Mouse Anti-Human CD184 (CXCR4) Clone 12G5, BD, 560936, 1:5<br>GARP Monoclonal Antibody (G14D9), FITC, eBioscience, 11-9882-41, 1:20<br>PE Mouse Anti-Human CD140a (PDGFRα) Clone αR1, BD, 556002, 1:20

	LEGENDScreen PE-Conjugated Human Antibody Plates; 1003 Biolegend, 700007
Validation	Anti-AP-2γ - IF<br>Validation status unknown; application validated by several publications including Chen D, Liu W, Zimmerman J, et al. The TFAP2C-Regulated OCT4 Naive Enhancer Is Involved in Human Germline Formation. Cell Rep. 2018;25(13):3591-3602.e5. doi:10.1016/j.celrep.2018.12.011.<br><br>Anti-Blimp1 (6D3) - IF<br>Validation reference - Kobayashi T, Zhang H, Tang WWC, et al. Principles of early human development and germ cell program from conserved model systems. Nature. 2017;546(7658):416-420. doi:10.1038/nature22812<br><br>Anti-Hand1 - IF<br>Validation reference - Loh KM, Chen A, Koh PW, et al. Mapping the Pairwise Choices Leading from Pluripotency to Human Bone, Heart, and Other Mesoderm Cell Types. Cell. 2016;166(2):451-467. doi:10.1016/j.cell.2016.06.011<br><br>Anti-Nanog - IF<br>Validated by supplier with more than 60 references.<br><br>Anti-Oct4 - IF<br>Validated by manufacturer during development.<br><br>Anti-Sox17 - IF<br>Validated by manufacturer and with more than 90 references.<br><br>CXCR4 APC - FACS<br>Routinely tested by manufacturer for flow cytometry.<br><br>GARP FITC - FACS<br>Tested by manufacturer for FACS applications.<br><br>PDGFRα PE - FACS<br>Routinely tested by manufacturer for flow cytometry.

Eukaryotic cell lines

Policy information about cell lines

Cell line source(s)	NANOS3-mCherry hESCs were obtained from the lab of Jacob Hanna (Weizmann Institute of Science). SOX17-GFP hESCs were obtained from the lab of Seung Kim (Stanford University). H9 NANOG-YFP cells were generated at Stanford University as described in Martin et al., 2019. BJC1, BJC3, and BIRc3 hiPSC lines were generated by the Sebastiano lab. H1 and H9 cell lines are standard and widely available embryonic stem cell lines.
Authentication	hIPSC cell lines (BJC1, BJC3, BIRc3) were validated by antibody staining and qPCR for known human pluripotent stem cell markers (OCT4, SOX2, NANOG). Genomic integrity was confirmed by karyotyping.
Mycoplasma contamination	Cells were routinely tested for mycoplasma contamination by PCR and/or immunofluorescence staining with DAPI.
Commonly misidentified lines<br>(See ICLAC register)	*Name any commonly misidentified cell lines used in the study and provide a rationale for their use.*

Flow Cytometry

Plots

Confirm that:

☒ The axis labels state the marker and fluorochrome used (e.g. CD4-FITC).

☒ The axis scales are clearly visible. Include numbers along axes only for bottom left plot of group (a 'group' is an analysis of identical markers).

☒ All plots are contour plots with outliers or pseudocolor plots.

☒ A numerical value for number of cells or percentage (with statistics) is provided.

Methodology

Sample preparation	hPSCs or their differentiated derivatives were dissociated using TrypLE Express (Gibco), were washed off the plate with FACS buffer (PBS + 0.1% BSA fraction V [Gibco] + 1 mM EDTA [Gibco] + 1% penicillin/streptomycin [Gibco]) and were pelleted by centrifugation (5 mins, 4 °C). Subsequently, cell pellets were directly resuspended in FACS buffer containing pre-diluted primary antibodies, thoroughly triturated to ensure a single cell suspension, and primary antibody staining was conducted for 30 mins on ice. Afterwards, cells were washed with an excess of FACS buffer and pelleted again, and this was conducted one

more time. Finally, washed cell pellets were resuspended in FACS buffer containing 1.1 µM DAPI (Biolegend), and were strained through a 35 µm filter prior to sorting.

Instrument	FACS was performed on a BD FACSAria II and CytoFLEX Flow Cytometer.
Software	FlowJo v10.6.2 was used to analyze flow cytometry data.
Cell population abundance	Cell population abundances are indicated in Figs. 1-3, S1, and S5.
Gating strategy	Gating strategy is identical to the one outlined in Loh et al., 2014, Cell Stem Cell, figure S2 subpanel K.

☒ Tick this box to confirm that a figure exemplifying the gating strategy is provided in the Supplementary Information.
